# Allopregnanolone-mediated GABAA-Rα4 function in amygdala and hippocampus of PMDD liver qi-invasion syndrome model rats

**DOI:** 10.18632/aging.204541

**Published:** 2023-02-23

**Authors:** Ya Sun, Mingzhou Gao, Dongmei Gao, Dan Chen, Jieqiong Wang

**Affiliations:** 1 Team of Research and Innovation Focusing on Emotional Diseases and Syndromes, Innovation Research Institute of Traditional Chinese Medicine, Shandong University of Traditional Chinese Medicine, Jinan 250355, Shandong, China; 2 Teaching and Research Office of Basic Theory of Traditional Chinese Medicine, College of Traditional Chinese Medicine, Shandong University of Traditional Chinese Medicine, Jinan 250355, Shandong, China; 3 Department of Graduate, Shandong University of Traditional Chinese Medicine, Jinan 250355, Shandong, China; 4 Scientific Research Achievements Transformation Department, Office of Academic Research, Shandong University of Traditional Chinese Medicine, Jinan 250355, Shandong, China

**Keywords:** ALLO, GABAA-Rα4, PMDD-LIS rat model, PROG, finasteride

## Abstract

Background: Premenstrual dysphoric disorder (PMDD) is a common mental health challenge among women of reproductive age. Allopregnanolone (3α, 5α-THP; ALLO) mediated functional alterations of GABAA receptors (GABAA-R) are involved in PMDD pathogenesis, however, the specific mechanism remains unknown. Therefore, we investigated the role of ALLO mediated GABAA-Rα4 in the pathophysiology of PMDD.

Purpose: We determined whether the pathogenesis of PMDD is associated with ALLO mediated GABAA-Rα4 expression changes in different brain regions.

Methods: Rat models of PMDD liver-qi invasion syndrome (PMDD-LIS) were established via the resident intruder paradigm. Behavioral changes of rats were assessed by aggressive behavior tests, EPM and OFT. The levels of progesterone and ALLO in serum as well as brain areas were determined by ELISA. Variations in GABAA-Rα4 levels in brain regions were assessed by immunofluorescence and RT-PCR. Medicated serum was used to interfere with rat hippocampal neurons, and changes in Cl^-^ current were recorded through electrophysiology.

Results: Premenstrual anxiety and irritability of PMDD-LIS patients can be simulated in PMDD-LIS rat models. Exogenous ALLO significantly improved the anxiety behaviors of PMDD-LIS rats. Changes in ALLO among different brain regions varied. GABAA-Rα4 expressions were low in the amygdala and abnormally high in the hippocampus, however, ALLO alleviated these deviations. Whole-cell patch clamp recording technique showed a weaker Cl^-^ current intensity of PMDD-LIS rats, reduced neuroinhibitory functions and increased Cl^-^ current intensity in the ALLO group drug serum intervention and enhanced emotional inhibition function.

Conclusion: We established that ALLO regulation of the GABAA-Rα4 subunit in the amygdala and hippocampus is involved in PMDD-LIS pathogenesis.

## INTRODUCTION

Premenstrual dysphoric disorder (PMDD) is a serious form of premenstrual syndrome (PMS), it’s a typical emotional disease, that is characterized by anxiety, depression, irritability, and other mental symptoms. It manifests in the late luteal phase and is accompanied by some physical symptoms that interfere with the quality of life in 3–5% of women in their reproductive years [1]. The occurrence of PMS/PMDD is dependent on recurrent menstrual cycles [2]. Elucidation of the role of progesterone (PROG) and its metabolite (allopregnanolone, ALLO) in PMS/PMDD has attracted great interest. At the moment, the changing characteristics of estrogen and progesterone in the menstrual cycle of PMS/PMDD patients have not been defined, and clinical research shows inconsistent results. Nonetheless, most studies have found aberrant levels of PROG and ALLO in PMS/PMDD patients’ luteal phase [3, 4]. Researches discovered that the concentrations of serum ALLO among PMDD patients in the luteal phase exhibits an inverted “U” distribution with the menstrual cycle, thus, negative emotions of patients gradually increase in the luteal phase to a maximum on the last day of the luteal phase (the first day of menstruation) [5], as a result, the negative emotion of PMDD patients is closely associated with fluctuations in ALLO levels in the luteal phase. The journal Progress in Neurobiology published an article titled “Allopregnanone and mood disorders” [5] in 2014, summarizing research findings from the previous two decades and concluding that the effect of ALLO mediated gamma-amino-butyric acid A receptor (GABAA-R) causes PMDD negative emotional symptoms. The first paper “Allopregnanone: State of the Art” published in the same journal concluded that ALLO is a key regulator of physiological function, and provided a new approach to treating neurodegenerative and psychiatric diseases.

The study found that the reactivity of PMDD patients to GABAA-R agonist benzodiazepine was reduced, implying that GABA is involved in the regulation of PMS [6]. A proton magnetic resonance spectroscopy study showed that the level of GABA in the cerebral cortex of PMDD patients during the menstrual cycle was significantly different from that of normal controls, indicating that the GABA system in the brain was involved in the pathogenesis of PMDD [7]. In recent years, the experiment of preparing PMDD rat model by PROG withdrawal method has confirmed that the PROG metabolite ALLO can directly bind to GABAA-R in the brain, which can not only affect its expression level, but also change its configuration, resist further activation, and finally reduce GABA mediated inhibition of the central axis [8]. It demonstrates that PROG and the GABAA-R subunit have feedback regulation, and research into the reconstruction of the GABAA-R subunit may provide a more effective target for neurosteroids. GABAA-R is a gated receptor of chloride channel. There has agonist benzodiazepine receptor on the α subunit, which can increase the frequency of Cl^-^ channel opening by promoting the binding of GABA to GABAA-R, resulting in more Cl^-^ influx [9]. Our previous serum pharmacology and patch clamp experiments found that the serum of PMS rat model of liver qi-inversion could lead to significant abnormality of GABAA-R mediated Cl^-^ current in primary cultured rat hippocampal neurons [10]. Thus, GABA mediated GABAA-R dysfunction may be one of the important targets of PMDD-LIS.

ALLO is a positive regulator of GABAA-R, which can be combined with GABAA-R α, β subunit binding opens ion channels [11, 12]. Animal experiments have proven its anti-anxiety effects (high concentrations in the amygdala) [13]. In PMS/PMDD patients, ALLO mediates the changes in GABAA-R functions, thereby causing external symptoms [14, 15]. Clinical experiments by Kask K [16] confirmed that PMDD patients and healthy control groups intravenously injected with ALLO had self-sedative effects. Bäckström T [5, 17] reported that PMDD patients have reduced sensitivities to ALLO, and elevated ALLO concentrations were associated with negative emotional symptoms. This result is inconsistent with positive regulation of GABAA-R in ALLO, we speculate this outcome might be associated with GABAA-R in the luteal phase of PMDD patients’ α4, β, δ up-regulation of expression. One study indicated that elevated GABAA-Rα4 levels in the hippocampus are correlated with high anxiety [18], upregulation of the α4 subunit leads to relative insensitivity of GABAA-R to anxiolytic effects of benzodiazepines that alters the dynamics of GABAA-R in the limbic circuit [19]. According to the above research results, the pathogenesis of PMDD-LIS is closely linked to the concentration of ALLO and the function change of GABAA-R mediated by ALLO.

The current international classification of PMDD is largely based on emotional and physical symptoms. Selective serotonin reuptake inhibitors (SSRIs) are very efficacious and the first line pharmacologic treatment options for PMS/PMDD. However, about 40% of PMDD women do not respond to SSRIs [20, 21]. Yonkers [22] found that sertraline treatment of PMDD significantly improved anxiety and depression, indicating that PMDD should be treated according to the classification. According to Chinese traditional medicine, PMDD is a typical emotional disease that belongs to the premenstrual emotional disorder, premenstrual breast swelling, premenstrual abdominal distension, and pain [23]. Based on an epidemiologic investigation of pathogenic mechanisms and dialectical theory of traditional Chinese medicine, PMDD is divided into liver qi-inversion and liver qi-depression syndromes [24]. This study mainly discussed the pathogenesis of PMDD -LIS.

At present, PMDD is widely recognized as a complex disease caused by mental neuroendocrine disorder. According to a 2013 review published in the American Journal of Psychiatry and research on the pathogenesis and therapeutic mechanism of PMDD, PMDD is closely related to specific brain regions in the center [25]. Through BOLD-fMRI technology, Accortt EE found that the left prefrontal lobe of PMDD patients had less activity than the right, which was consistent with the menstrual related anxiety displayed in the quality stress model [26]. In 2013, Gingnell M found that the significant increase of adverse emotional stimulation in PMDD women was in the prefrontal cortex. Jeong HG found that the concentration of gray matter in hippocampus cortex of PMDD patients was significantly increased, while that in parahippocampal cortex was significantly decreased [27]. The right amygdala of PMDD patients with high trait anxiety score in luteal phase is more reactive, indicating that the amygdala of PMDD patients is more easily activated [28]. At the same time, patients with PMDD have stronger response to the stimulus of negative emotional words in the premenstrual amygdala nucleus, indicating that patients with PMDD have enhanced treatment of negative emotions in the premenstrual period [29]. Zhang YM found that serum PROG, ALLO, GABA and other hormones in the luteal phase of PMDD-LIS patients were unbalanced [30], which was also associated with levels of hormones and neurotransmitters in specific brain regions and abnormal expressions of their receptors [31]. According to the literature, the prefrontal cortex, amygdala and hippocampus are the main brain regions for PMDD.

Based on the above studies, the pathogenesis of PMDD-LIS is closely related to the functional changes of prefrontal cortex, amygdala, hippocampus and other related brain regions, and is closely related to progesterone and ALLO in related brain regions, but its specific pathological mechanism has not been clarified. Therefore, this study took the PMDD-LIS rat model as the carrier to explore the regulatory effect of PROG and ALLO on GABAA-R in specific brain regions.

## MATERIALS AND METHODS

### Animal preparation

Specific pathogen free (SPF) grade female Wistar rats 300 (6–8 weeks old, 180–220 g) were used in this study, SPF female Sprague Dawley (SD) rats 60 (160–200 g), animal license No: SCXK (Beijing) 2016–0006. All animals were purchased from the Charles River company (Beijing, China), and housed at 21 ± 2°C and 45 ± 10% relative humidity under a 12:12 h light/dark cycle with food and water available ad libitum. The animals were habituated to maintenance conditions for 1 week and handled daily to eliminate the human factor.

### Establishment of PMDD-LIS rat models

**Figure 1:**
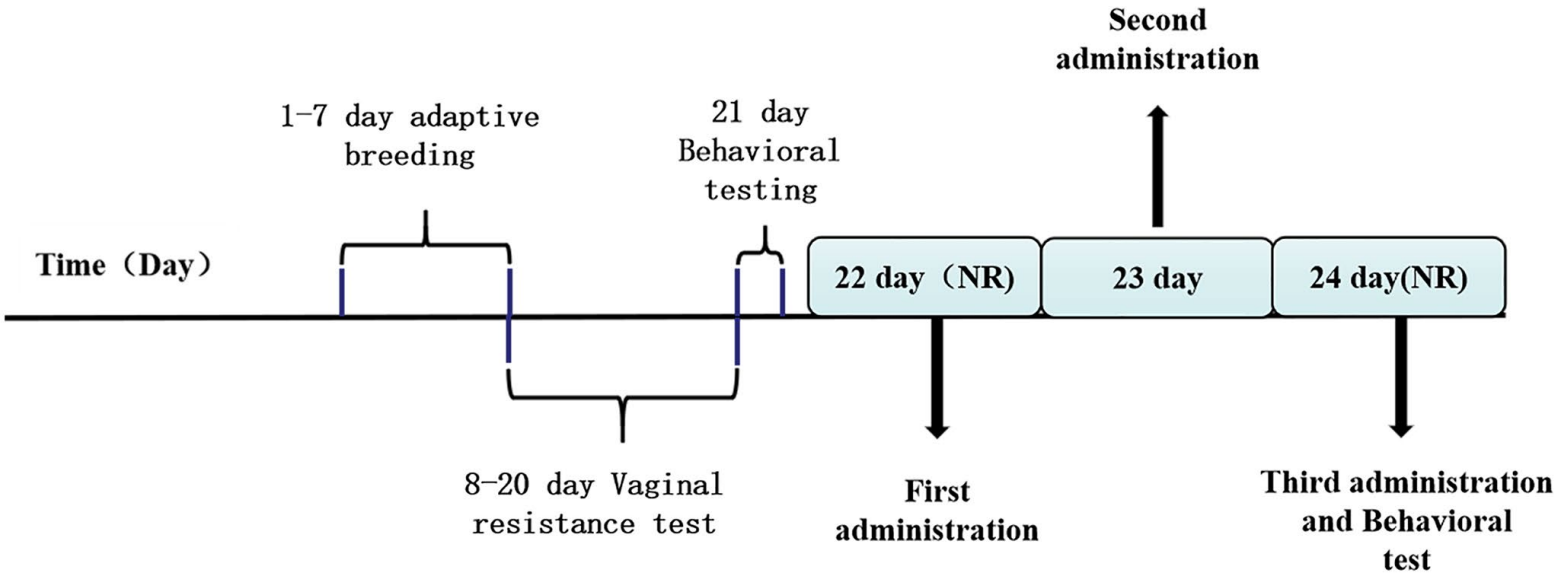
The drug management and behavioral testing schedule.

A typical rat oestrus is divided into four phases: non-receptive phase (3 days, including metoestrus, dioestrus I, and dioestrus II) and receptive phase (1-day, pro-oestrus/oestrus) [32]. Ovulation occurs in the receptive phase. After one week of adaptive feeding, the estrous cycles of normal non-pregnant rats were determined by the vaginal epidermal cell resistance test (Figure 1). The monitoring range of the resistance value of the rat estrus cycle detector is 0–19.9 KΩ, best detection time is 13:00–15:00. If the resistance value ≥3 KΩ is in the receptive phase, resistance value <3 KΩ is in the non-acceptance period. The estrus cycle of rats is generally 4–5 days, and it should be monitored at least for two consecutive cycles. Measuring method of vaginal electrical resistance: turn on the power switch of the rat pregnancy analyzer, check the relevant conditions of the equipment, and confirm that the initial value of the equipment is 19.90 KΩ, clean the vaginal probe with normal saline and place it aside for future use. Grasp the rat with the left hand to make it supine, gently insert the vaginal probe into the rat vagina, and record the data after the value is stable. Rats with estrous cycle regularity were screened for single cage feeding and subsequent experiments [33].

Adult female SD rats weighing less than 50 g were selected as invasive rats. Then, bilateral ovaries were removed to reduce the impact of the estrous cycle on their emotions. Gentamicin sulfate (Kaifeng, H41024114, China) was intramuscularly injected for 3 days post operation at 40 mg/mL to prevent infections. Rats were allowed to recover for at least one week after operation, after which a residential invasion experiment was conducted.

Selected rats with estrous cycles were subjected to a residential invasion experiment, and normal as well as PMDD-LIS rat models were selected according to the aggressive behavior fighting score. The aggressive behavior test was performed in the non-acceptance period of experimental rats, and the test time was 14:00–17:30. This was done in the breeding environment of residential rats. At least 30 min before the start of the experiment, invasive rats were moved to the residential rat environment to adapt to the new environment. Operation steps were performed as previously described [34, 35]. Resident rats and their cages were placed under a camera, then, the invasive rat was placed into the cage for 10 min. Afterward, the invasive rat was taken out and the comprehensive aggression score of resident rats counted. The formula used for establishing the comprehensive aggression score was: (Number of attacks + 0.2) × (Attack time(s) + Number of bites + 0.2) × Jump time(s).

Animals were ranked from high to low according to aggressive behavior scores. The top 30% animals were randomized into the PMDD-LIS model group (Model group), PMDD-LIS + ALLO group (ALLO group), and the PMDD-LIS + 5α reductase inhibitor group (Finasteride group), while the latter 30% were assigned into the normal group, and the rest of the animals were eliminated [36].

### Drug treatment regimen

The first administration is in the interval phase II (Figure 1). Animals were injected (intraperitoneally) with either ALLO (10 mg/kg in sesame oil, Med Chem Express, 24259, USA), finasteride (50 mg/kg in sesame oil, Med Chem Express, 28863, USA) once each morning (between 9:00 AM and 10:00 AM) over a 48-h period for a total of three injections during this period. Control group and model group animals were given the same number of injections of vehicle (sesame oil). Animals were tested 3 to 4 h after the final hormone injection. During drug treatment, the vaginal resistance test was performed to master the menstrual cycles of rats.

### Behavioral experiment

The elevated plus maze (EPM) protocol was performed as previously described [37]. Experimental rats were placed in a central area with their heads facing the open arms, after which the activities of experimental rats were recorded within 300 s. The times of entering the open arm (OE), times of entering the closed arm (CE), time in the open arm (OT, s), and time in the closed arm (CT, s) were recorded. The percentage of entering the open arm (OE%) and the percentage of open arm residence time (OT%) were calculated from the above indicators. The calculation formula was: OE% = OE/(OE + CE) × 100%, OT% = OT/(OT + CT) × 100%. After the test of each rat, the equipment was cleaned with 75% alcohol.

The Open-Field Test (OFT) protocol was performed as previously described [38]. The 100 × 100 cm open field was divided into 9 squares on average. Experimental rats were placed in the central area of the open field box, timed, and recorded. The Smart3.0 (Shanghai Xinruan, XR-XZ301, China) experimental animal behavior video acquisition and analysis system were used to record the total distance of rats’ movement, the number of times they entered the central area (more than 50% of rats’ bodies entered), and duration in the central area within 360s. The equipment was cleaned with 75% alcohol after the assessment of each rat.

All behavioral experiments were conducted in duplicates, during the dioestrus II period before and at 3–4 h after the last administration. Concurrently, vaginal resistance of resident rats was tested daily to determine the estrous cycle, and rats with irregular estrous cycles removed.

### Sample preparation

Behavioral experiments were performed at 3–4 h after the last administration. Materials (serum and specific brain areas) were immediately obtained after completion of the experiments. Respiratory anesthesia (isoflurane, RWD Life Science, 20190120, China) was induced to ensure that rats were in a coma during the collected process.

Serum sample preparation: After acclimatization at room temperature for 30 min, 5 ml of blood sample was extracted from the abdominal aorta and centrifuged using a high-speed centrifuge (Eppendorf, 5418, Germany) at 4°C, 3500 rpm for 10 min. Then, the supernatant was obtained and stored at −8°C for subsequent assaying of hormone activities and for whole cell patch clamp experiments.

Brain sample preparation: After blood extraction, rats were decapitated and their brains extracted. The prefrontal cortex, hypothalamus, amygdala, and hippocampus were surgically removed, separated on ice, weighed, and recorded for Western blotting and RT-qPCR analysis. Three rats in each group were perfused with 500 ml, 0.9% normal saline, and 150 ml 4% paraformaldehyde (Dingshengxin, 69120900, China) after anesthesia. Whole brains were resected and fixed in 4% paraformaldehyde for immunofluorescence.

### Assessments of PROG and ALLO levels

Serum and brain area levels of PROG and ALLO were determined using the enzyme-linked immunosorbent assay (ELISA) kit (CUSABIO, C0145080175, China; Kejing Biology, 201901, China), in accordance with the manufacturers’ instructions. Absorbance was read at 450 nm using the microplate reader (Shanpu, SP-MAX2300A2, China). Each sample was tested in duplicates to reduce errors.

### Immunofluorescence technique

Brain tissues were fixed for at least 24 h, dehydrated, transparent, and treated into wax blocks for preservation. The wax blocks were evenly sliced into 3 μm thick sections using a microtome for fixation. Then, dewaxing, repair, membrane breaking, and blocking were carried out according to specific steps, followed by incubation with antibodies (dilution of the first antibody at 1:300, dilution of the secondary antibody at 1:400). Later, autofluorescence quenching, 4’, 6-diamidino-2-phenylindole (DAPI) counterstaining of nuclei, and finally sealing was performed. The slides were placed under a fluorescence microscope (NIKON, D-Eclipse C1, Japan) for observation and image acquisition (Cy3 excitation wavelength 510–560, 590 nm wavelength emitting red light) [39].

### Reverse transcription real-time quantitative polymerase chain reaction (RT-qPCR)

Total RNA was extracted from the prefrontal lobe, hypothalamus, amygdala, and hippocampus using the RNA Extraction Kit (7E303H9, Vazyme, China) and transcribed into cDNA using the reverse transcription kit (Vazyme, R232-01, China). RT-qPCR was performed using an Agilent Real-time fluorescent quantitative PCR instrument (AriaMx) and SYBR Green (New England Biolabs, e3005l, USA). Each sample was run in triplicates. Relative gene expressions were calculated using the 2^(−ΔΔCt) method. The internal reference primers (Table 1) were synthesized by Sangon Biotech (Shanghai, China).

**Table 1:** Sequences of the target gene and internal reference gene.

**Primers**	**Primer sequences**
GAPDH	F: TCTCTGCTCCTCCCTGTTCT’
	R: TACGGCCAAATCCGTTCAC
GABAA-Rα4	F: GAAACCACTCCTAAGGCCCACT’
	R: GCGATGCGGCAGACGAAA

### Whole-cell patch clamp

The skins of newborn SD rats were disinfected with 75% ethanol, quickly excised, their hippocampus were removed, and hippocampal neurons cultured. After seven days of culture, they were treated with serum-containing drugs, and changes in Cl^-^ currents were recorded with a whole-cell patch clamp.

The voltage stimulation scheme of the whole cell patch clamp recording GABA current was as follows [40]: after formation of the whole cell seal, the cell membrane voltage was clamped at −70 mV and the cell surface injected with 30 μM GABA, 10 μM Str, and 300 nm TTX. The peak value of the current was recorded in the gap-free mode. The test data was collected using the EPC-10 amplifier (HEKA, EPC-10, Germany) and stored in the patchmaster (HEKA, Patchmaster, Germany; HEKA, GraphPad, Germany) software.

A capillary glass tube (Sutter Instruments, BF150-86-10, USA) was drawn into a recording electrode with a microelectrode puller (Sutter Instruments, P97, USA). A microelectrode manipulator (MP285, Sutter Instruments) was set under an inverted microscope (Olympus, IX71, Japan) to contact the recording electrode with the cell, and negative pressure suction was applied to form a GΩ seal. After GΩ sealing, fast capacitance compensation was performed. Then, the negative pressure was continuously applied to break the cell membrane to form the whole-cell recording mode. Finally, slow capacitance compensation was performed and membrane capacitance and series resistance were recorded. No leakage compensation was given.

### Statistical analysis

Data are shown as mean ± standard deviation (SD). Graph pad prism 8.0.1 software was used to develop graphs while the IBM SPSS statistics V21 software was used for statistical analyses. Comparisons of means among groups was performed by one-way analysis of variance (ANOVA). The significance levels were set at: ^*^p < 0.05, ^**^p < 0.01.

## RESULTS

### Anxiety-like behaviors of model rats and ALLO intervention

**Figure 2:**
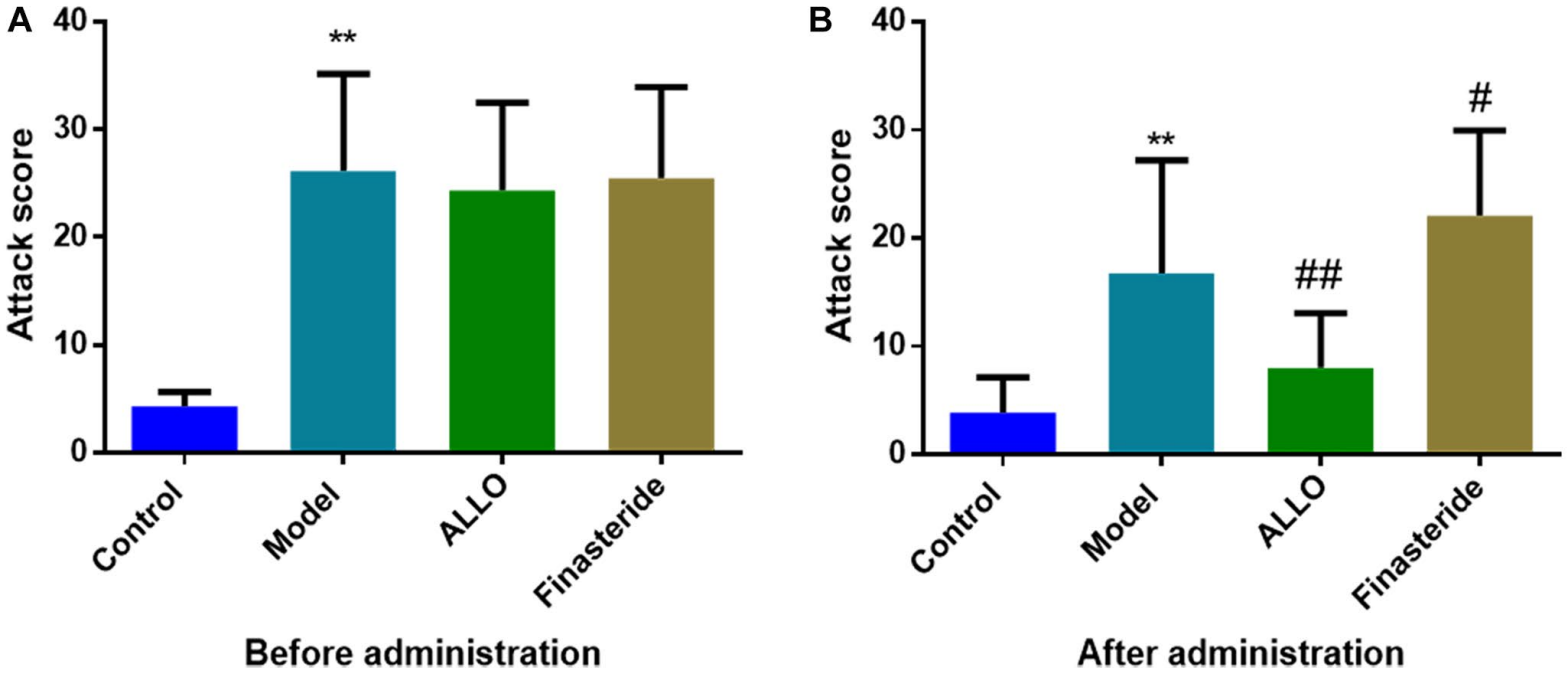
**Effects of ALLO on aggressive behaviors of PMDD-LIS rat models.** (**A**) A score of aggressive behaviors before administration. (**B**) A score of aggressive behaviors after administration. Compared with the normal group: ^*^p < 0.05, ^**^p < 0.01; compared with the model group, ^#^*p* < 0.05, ^##^*p* < 0.01.

**Figure 3:**
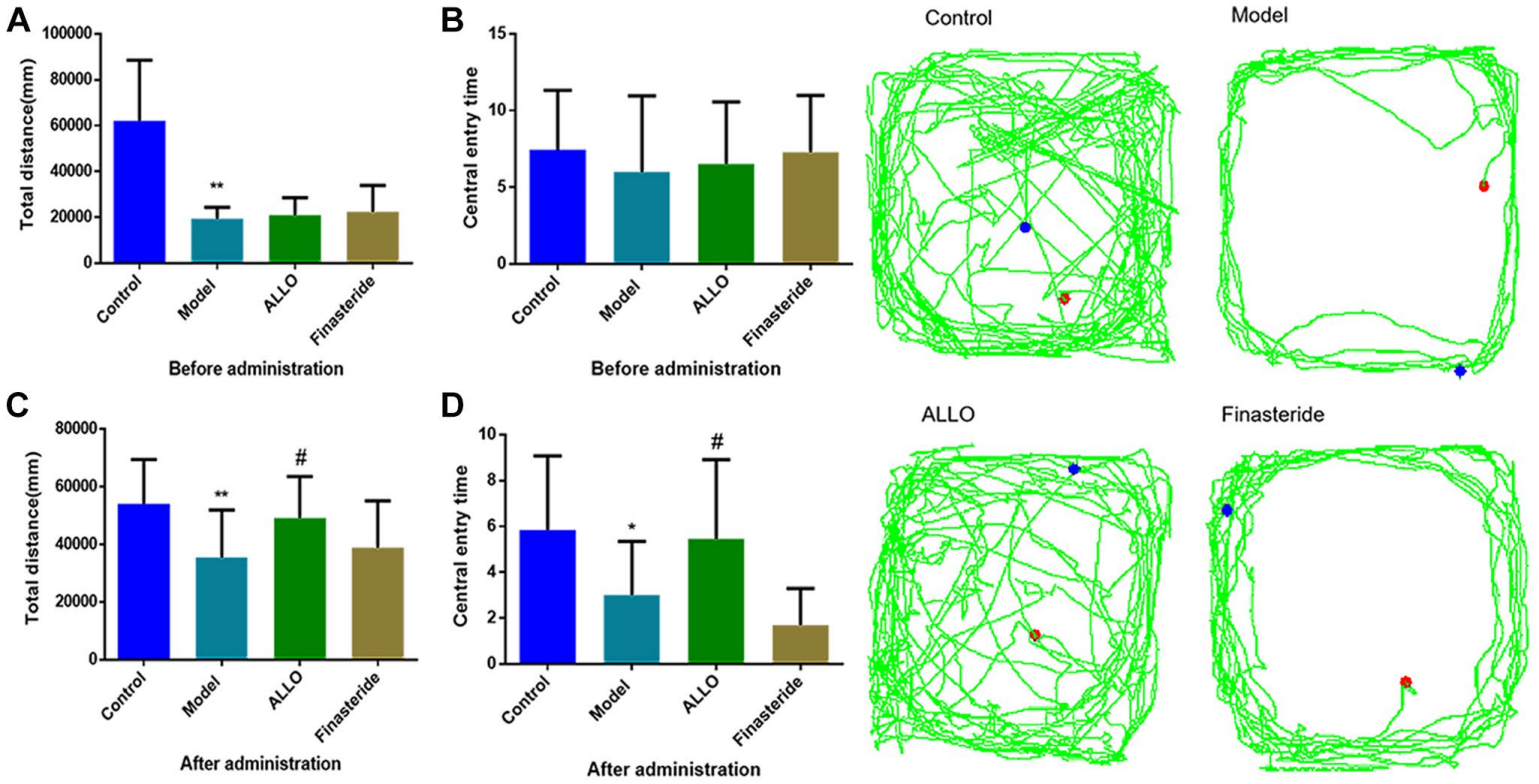
**Effects of exogenous ALLO on OFT in PMDD-LIS rat models.** (**A**) Total distance before administration. (**B**) Times of entering the central area before administration. (**C**) Total distance after administration. (**D**) Times of entering the central area after administration. Compared with the normal group: ^*^p < 0.05, ^**^p < 0.01; compared with the model group, ^#^*p* < 0.05, ^##^*p* < 0.01.

**Figure 4:**
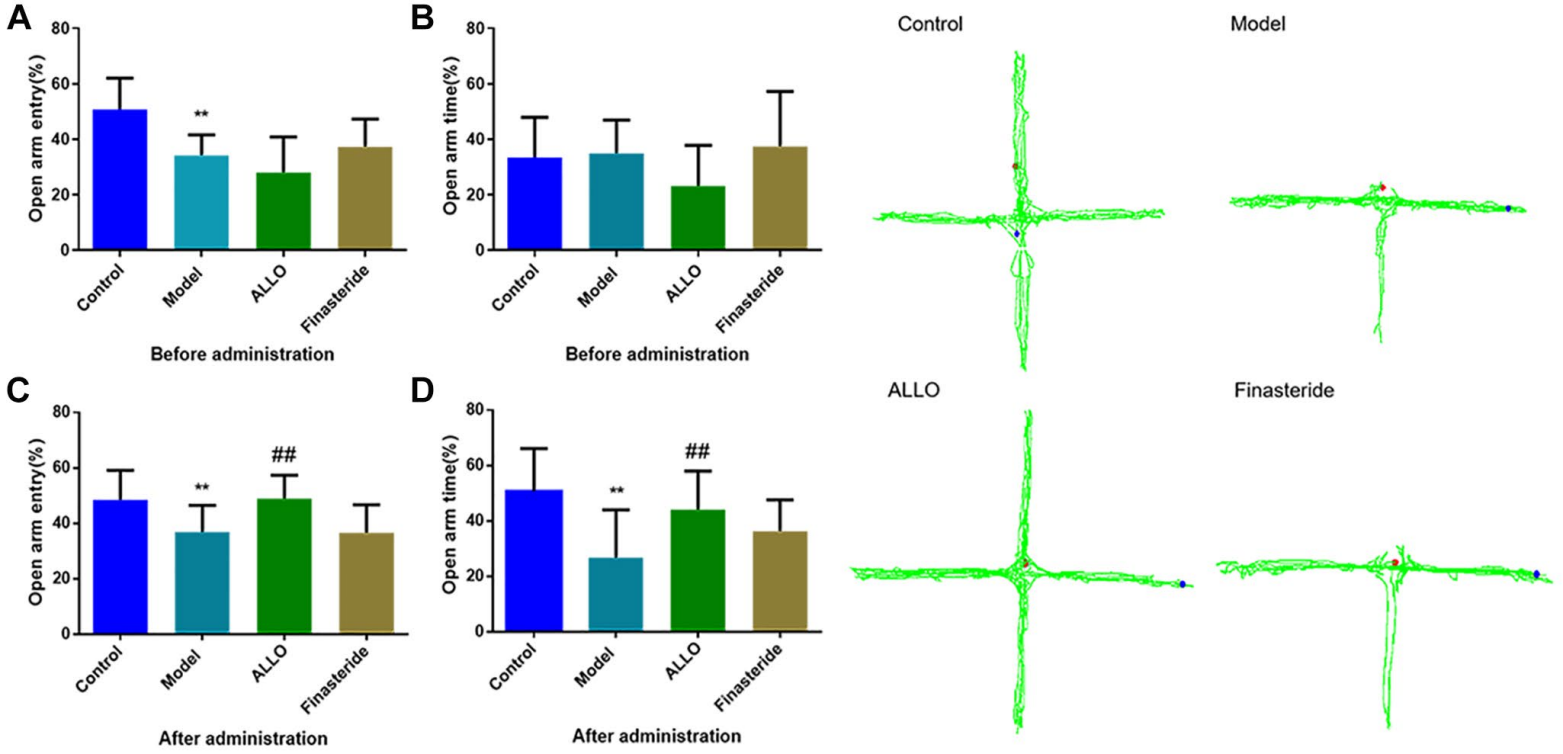
**Effects of exogenous ALLO on EPM in PMDD-LIS rat models.** (**A**) The OE% of rats before administration, (**B**) The OT% of rats before administration, (**C**) The OE% of rats after administration, (**D**) The OT% of rats before administration. Compared with the normal group: ^#^*p* < 0.05, ^##^*p* < 0.01; compared with the model group, ^#^*p* < 0.05, ^##^*p* < 0.01.

Behavioral analyses showed that aggressive behavior scores of rats in the non-acceptance period were significantly high (*p* = 0.000, Figure 2A). The total distance of OFT and the number of entries into the central area were significantly reduced (Figure 3A). The OE% (*p* = 0.000, Figure 4A) and OT% (*p* = 0.000, Figure 4D) in the EPM were significantly low. This shows that after modeling, rats had higher anxiety levels in the non-acceptance period, consistent with clinical manifestations of anxiety and irritability in patients with liver-qi invasion syndrome of PMDD. This model rat can be used as a PMDD-LIS model for the follow-up experiments.

After drug treatment, aggressive behaviors of the ALLO group (Figure 2B) significantly decreased (*p* = 0.001). Besides, finasteride enhanced the aggressive behaviors of PMDD-LIS rats (*p* = 0.050, Figure 2B). In the OFT experiment (Figure 3A–3D), after medication, the total distance (*p* = 0.050) and the number of times (*p* = 0.050) of the ALLO group’s movement were reversed to the normal level. There were no significant changes in the total distance of exercise (*p* = 0.050) and the number of times of entering the central area of the finasteride group. In the EPM analysis (Figure 4A–4D), following exogenous ALLO treatment, the OE% (*p* = 0.003) and OT% (*p* = 0.004) of rats were significantly improved, and their exploration ability was enhanced.

### Levels of PROG and ALLO in serum and brain regions of PMDD-LIS rats

**Figure 5:**
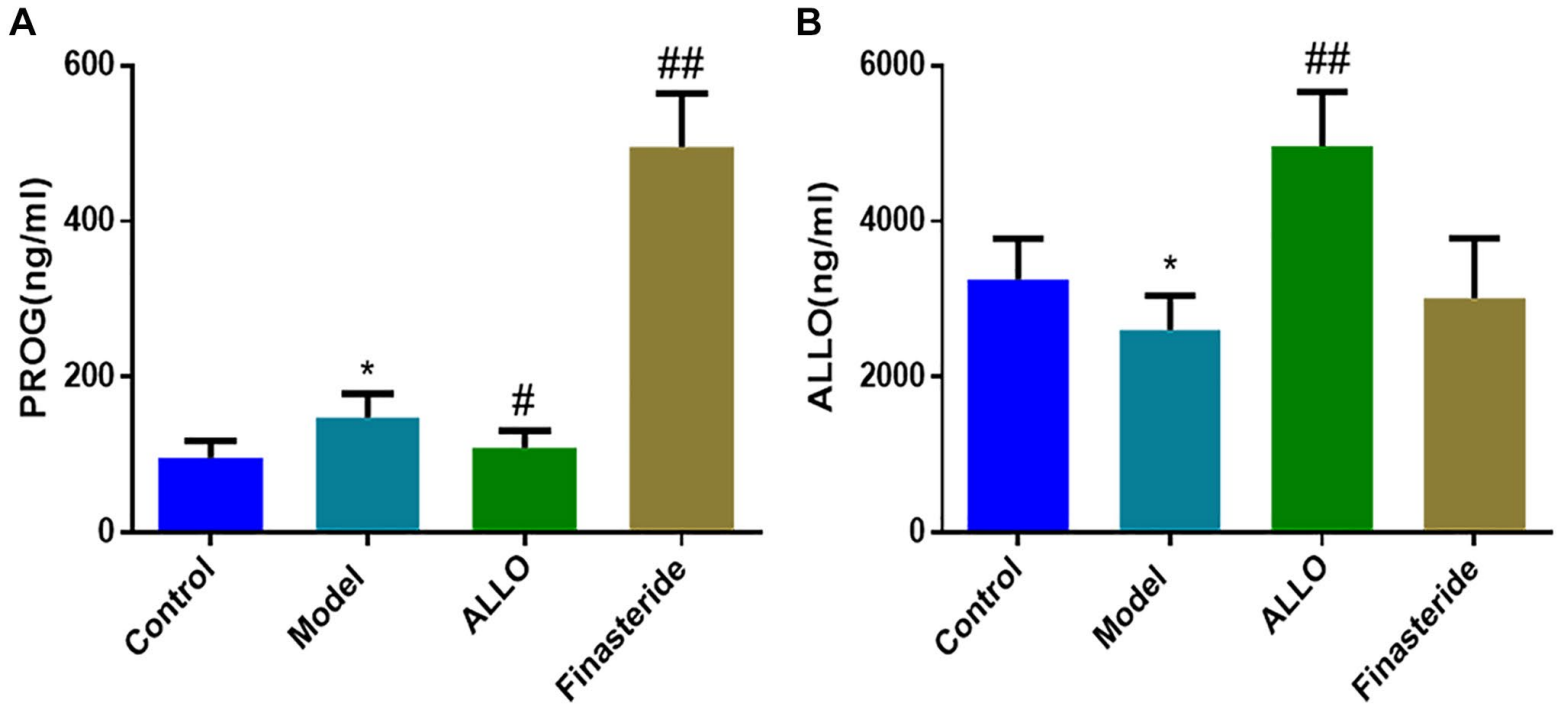
**Effects of exogenous ALLO on serum PROG and ALLO level in rats with PMDD-LIS.** (**A**) PROG content in rat serum. (**B**) ALLO content in rat serum. Compared with the normal group: ^*^p < 0.05, ^**^p < 0.01; compared with the model group, ^#^*p* < 0.05, ^##^*p* < 0.01.

In Figure 5, serum levels of PROG in the model group were higher than those of the normal group (*p* = 0.029), while ALLO levels were low (*p* = 0.045). The PROG levels in the ALLO group and model groups were comparable (*p* = 0.180), however, ALLO levels in the ALLO group were significantly higher than those of the model group (*p* = 0.000). After finasteride treatment, PROG levels were “cliff-shaped” (*p* = 0.000), indicating successful inhibition of PROG transformation into ALLO.

**Figure 6:**
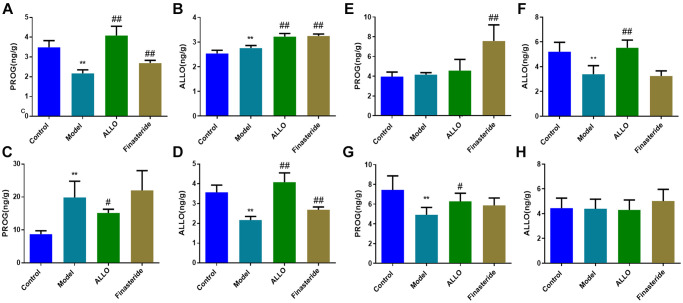
**Effects of exogenous ALLO on PROG and ALLO contents in the brain of rats with PMDD-LIS.** (**A**, **B**) Levels of PROG and ALLO in the prefrontal cortex, respectively. (**C**, **D**) Levels of PROG and ALLO in the hypothalamus, respectively. (**E**, **F**) Levels of PROG and ALLO in the amygdala, respectively. (**G**, **H**) Levels of PROG and ALLO in the hippocampus, respectively. Compared with the normal group: ^*^p < 0.05, ^**^p < 0.01; compared with the model group, ^#^*p* < 0.05, ^##^*p* < 0.01.

The concentration of PROG in the prefrontal cortex (*p* = 0.000) and hippocampus (*p* = 0.000) of PMDD-LIS rats were lower than those of the normal group (Figure 6), whereas the concentration of PROG in the hypothalamus increased (*p* = 0.000). Exogenously administered ALLO significantly increased PROG content in the prefrontal cortex (*p* = 0.000) and hippocampus (*p* = 0.000) of PMDD-LIS rats and reduced PROG content in the hypothalamus. The content of ALLO in the prefrontal cortex of rats (*p* = 0.001) was higher than those of the normal group, while ALLO levels in the hypothalamus and amygdala were suppressed (*p* = 0.000). However, exogenous ALLO significantly increased ALLO level in the prefrontal cortex, hypothalamus, and hippocampus of PMDD-LIS rats (*p* = 0.000).

### Levels of GABAA-Rα4 in the brains of PMDD-LIS model rats

**Figure 7:**
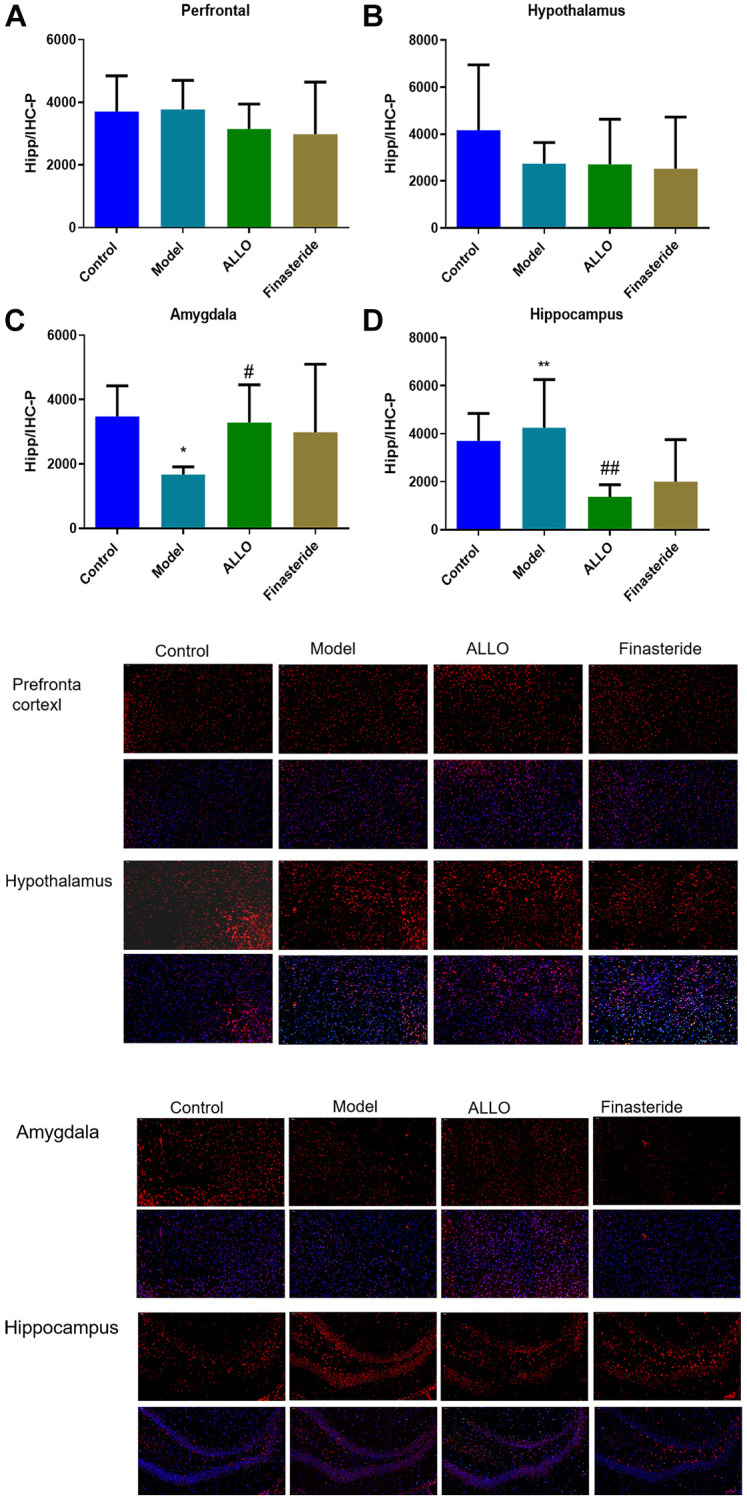
**Expression of GABAA-Rα4 in brain regions of rats in each group.** Red labeled GABAA-Rα4, nuclei were labeled with DAPI (blue). (**A**) α4 subunit expressions in the prefrontal cortex of rats. (**B**) α4 subunit expression in the hypothalamus of rats. (**C**) α4 subunit expression in the amygdala of rats. (**D**) α4 subunit expressions in the hippocampus of rats. Compared with normal group: ^*^p < 0.05, ^**^p < 0.01; compared with the model group, ^#^*p* < 0.05, ^##^*p* < 0.01.

Immunofluorescence analysis showed that compared with the normal group, α4 subunit expressions were suppressed in the amygdala of PMDD-LIS rat models (*p* = 0.019), but elevated in the hippocampus (*p* = 0.001). After treatment with exogenous ALLO, expression of the α4 subunit in the amygdala of PMDD-LIS rat models was elevated (*p* = 0.035), but was low in the hippocampus (*p* = 0.000; Figure 7).

**Figure 8:**
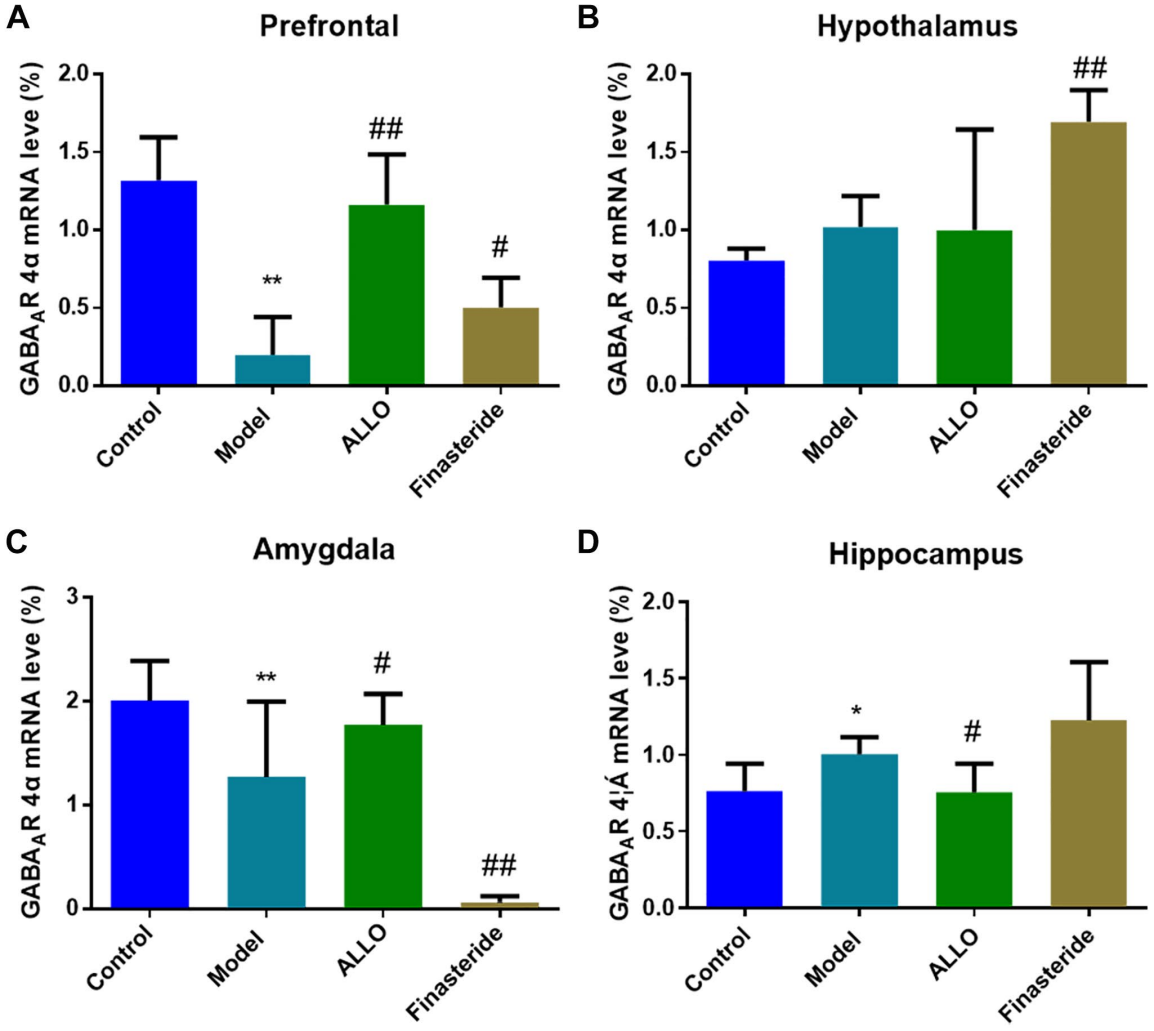
**Effects of ALLO on GABAA-R-α4 mRNA expressions in the brains of PMDD-LIS rats.** (**A**) α4 subunit mRNA expression in the prefrontal cortex of rats. (**B**) α4 subunit mRNA expression in the hypothalamus of rats. (**C**) α4 subunit mRNA expression in the amygdala of rats. (**D**) α4 subunit mRNA expression in the hippocampus of rats. Compared with the normal group: ^*^p < 0.05, ^**^p < 0.01; compared with the model group, ^#^*p* < 0.05, ^##^*p* < 0.01.

RT-PCR analysis revealed decreased levels of α4 subunit expression in the prefrontal cortex (*p* = 0.000) and amygdala (*p* = 0.001) of PMDD-LIS rats, compared with the normal group. However, α4 subunit expressions were elevated in the hippocampus. Exogenous treatment with ALLO elevated α4 subunit expressions in the prefrontal cortex (*p* = 0.000) and amygdala (*p* = 0.021), but decreased these expressions in the hippocampus (*p* = 0.032; Figure 8).

### Whole-cell patch clamp technique outcomes

**Figure 9:**
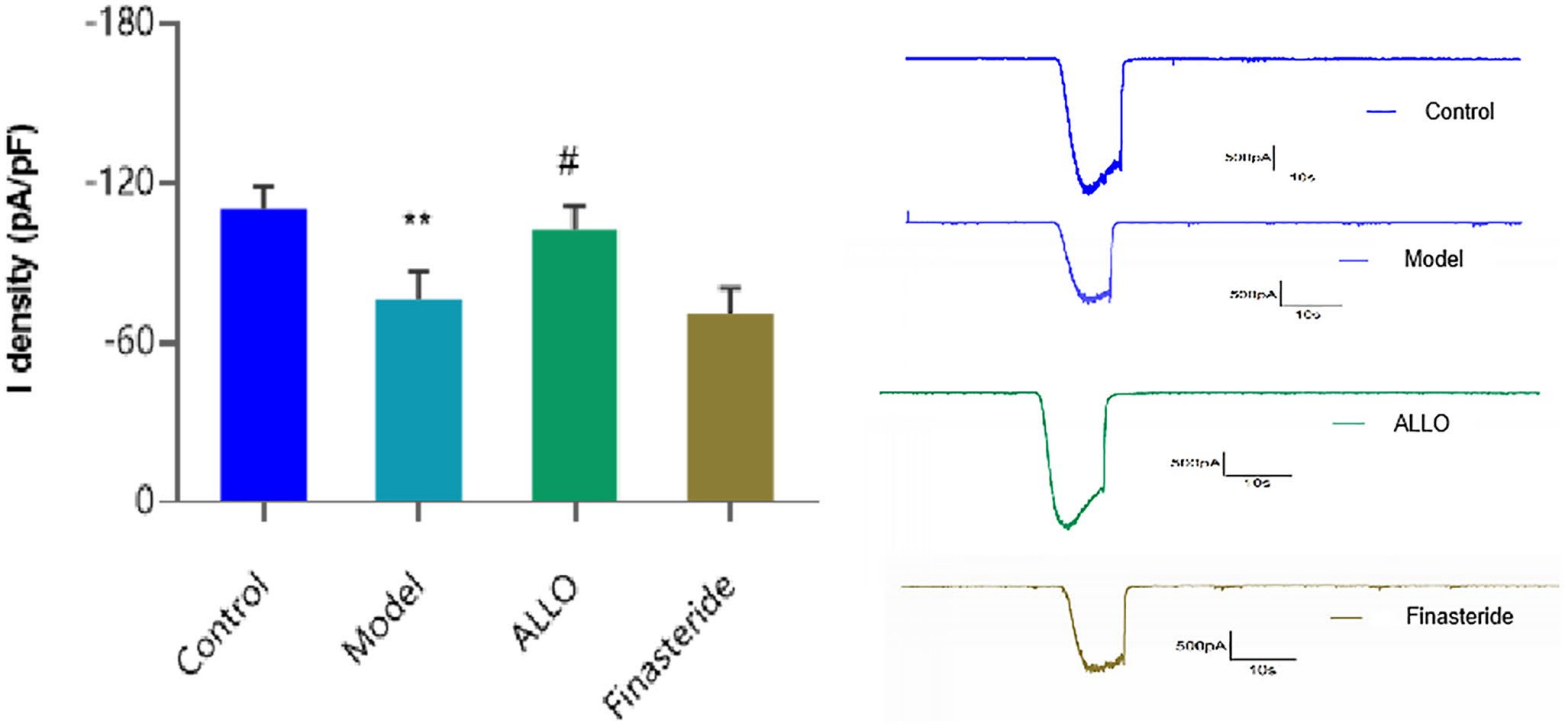
**Cl^-^ current density in hippocampal neurons.** Compared with the normal group: ^*^p < 0.05, ^**^p < 0.01; compared with the model group, ^#^*p* < 0.05, ^##^*p* < 0.01.

Current densities of hippocampal neurons in each group are shown in Figure 9. After treatment with serum from PMDD-LIS rats, there was a marked decrease in current density of Cl^-^ (*p* = 0.004), while the current density of Cl^-^ in hippocampal neurons incubated with ALLO-containing was serum increased (*p* = 0.028).

## DISCUSSION

Defense behavior patterns of humans and non-human mammals are similar [41]. Anger and anxiety of rats can effectively simulate the irritability of clinical PMDD-LIS patients. The common method for establishing PMDD rat models is resident intruder paradigm (RIP). In this experiment, rats with normal periodic release of hormones were selected as experimental objects, and the cycle-dependent aggressive behaviors (irritability and anger) were elicited by introducing an intruder rat into a resident rat cage [36, 42]. The PMDD-LIS rat models exhibited aggressive behaviors in the non-receptive phase (similar to premenstrual period of women), therefore, the resident invasive animal model was proven to be a reliable PMDD-LIS rat model for studies [32].

In animal ethological experiment, we found that the attack scores of model group rats were higher, and the total distance of OFT and the number of open arm explorations of EPM were less than control group. After drug intervention, in ALLO group, the scores of aggressive behavior, the total distance of OFT, the time to enter the central area, OE% and OT% all increased significantly; in the finasteride group, OE%, OT% and aggressive behavior scores increased significantly. The above behavioral experimental results show that ALLO can improve the exploration behavior of rats and the anxiety and irritability of PMDD-LIS rats, which is consistent with the experimental results of Frye [43]. The behavioral experimental results show that the anxiety-like behavior of PMDD-LIS rat model was closely related to ALLO, which provided preliminary evidence for the relationship between PMDD - LIS and ALLO.

Further, PROG levels in the serum of PMDD-LIS rats in the non-receptive phase were increased and ALLO levels were decreased, which is consistent with the changes in PROG levels in the peripheral serum of PMDD women in the late luteal phase [44]. After ALLO treatment, PROG levels in serum of PMDD-LIS rats were decreased and ALLO levels were markedly increased. After intraperitoneal injection of finasteride, the content of PROG in serum increased sharply, which indicated that finasteride inhibited the transformation of PROG into ALLO.

A large number of studies have shown that PMDD is closely related to specific areas of the brain center. In this study, compared with the control group, PMDD-LIS rats had lower PROG levels in the prefrontal cortex, amygdala and hippocampus, but higher PROG levels in the hypothalamus, which was reversed by exogenous ALLO therapy. The concentration of ALLO in the hypothalamus and amygdala in the prefrontal cortex of PMDD-LIS rats decreased, exogenous ALLO significantly increased the content of ALLO in the hypothalamus and amygdala of PMDD-LIS rats. Immunofluorescence and RT-PCR analyses showed that the pathogenesis of PMDD-LIS was related to the abnormal expression of GABAA-Rα4 in the prefrontal cortex, amygdala, and hippocampus, and the mechanism was different among different brain regions. We found that the level of ALLO in the amygdala of PMDD-LIS rats was lower than those of the control group, and GABAA-Rα4 in ALLO group increased expression. The expression of the GABAA-Rα4 subunit in the hippocampus of PMS-LIS rats was markedly elevated, similar to the increase of GABAA-Rα4 subunit expression in the hippocampus of rats with anxiety as reported in [18, 19]. Exogenous ALLO reduced the expression of the GABAA-Rα4 subunit in the hippocampus of PMDD rats. The increase of α4 subunit can lead to the increase of anxiety in rats, which is consistent with the reports in the literature [17–19].

GABA is a gated receptor for chloride ion channels, and there are GABA receptor sites in β subunit, when GABA binds to it, the Cl^-^ channel opens and Cl^-^ flows in, which makes nerve cells hyperpolarize, resulting in inhibitory effect. The α4 subunit has the agonist benzodiazepine receptor, when benzodiazepine is combined with it, it cannot open the Cl channel, but it increases the frequency of opening the Cl^-^ channel by promoting the combination of GABA and GABAA-R, leading to more Cl^-^ influx. In our previous serum pharmacology and patch clamp experiments, we found that the serum of PMS rats with liver-qi deficiency syndrome could cause abnormal Cl^-^ current in primary cultured hippocampal neurons of rats. The PMDD-LIS rat serum was used to treat hippocampal neurons from the rats, results showed that this intervention decreased the Cl^-^ current density, and reduced its neuroinhibitory function, which was consistent with anxiety and anger behaviors observed in model group rats. According to the literature, at low concentration, ALLO acts as a positive allosteric regulator of GABAA-R, but at high concentration, it directly activates the receptor in the absence of GABA [45]. The levels of GABA and ALLO in serum of rats with PMDD-LIS were low, GABAA-R regulation was weak, and Cl^-^ decreased, increased anxiety in rats. After drug interventions, the concentration of ALLO and GABA in the serum of ALLO group rats increased, the density of Cl^-^ current increased, and the anxiety of rats improved. Differences in outcomes between the finasteride group and the model group were insignificant. The above experimental results indicate that ALLO is involved in the pathological process of PMDD-LIS, possibly by mediating the function of GABAA-R and influencing cl channel.

The concentration of PROG in peripheral serum of rats increased while ALLO levels were decreased. Moreover, PROG and ALLO levels in the prefrontal cortex, amygdala, and hippocampus brain regions of the affected rats changed. GABAA-Rα4 gene expressions in the hippocampus were upregulated and decreased in the amygdala. Consequently, PMDD-LIS model rats showed aggression, anxiety, and other behaviors.

## CONCLUSIONS

The pathogenesis of PMDD-LIS was discussed in this study, ALLO can mediate the abnormal expression of GABAA-R-α4 in different brain regions (increased in hippocampus, decreased in amygdala), causing aggressive and anxious behavior in rats, which provides favorable evidence for the relationship between PMDD-LIS and ALLO.
